# Repair of cervicothoracic skin defects with extra-long transverse cervical flaps by stepwise pressure packing in children: a technical innovation

**DOI:** 10.3389/fped.2023.1269695

**Published:** 2023-11-21

**Authors:** Liangliang Kong, Jiageng Xiong, Yi Ji, Jie Cui, Jianbing Chen, Weimin Shen

**Affiliations:** Department of Plastic Surgery, Children's Hospital of Nanjing Medical University, Nanjing, China

**Keywords:** transverse cervical flap, skin expansion, stepwise pressure packing, children, neck defect

## Abstract

**Objective:**

To investigate the clinical effect of prolonging predilated transverse cervical flap with stepwise pressure packing for neck and chest lesions in children.

**Methods:**

A retrospective review of children with large cervicothoracic lesions admitted to our department from January 2011 to June 2021 was conducted to compare stepwise pressure packing with normal dressing in the surgical method of transverse cervical pedicled flaps after expansion. Among 58 included children, 22 (14 males and 8 females) were allocated to the extended and expanded transverse cervical flap with stepwise compression dressing group, and 36 (19 males and 17 females) to the transverse cervical flap group. The causes of skin defects were: scars (37 cases) and giant nevus (21 cases). The course of the disease ranged from 0.5 to 8 years. The two groups were compared in terms of child satisfaction, the occurrence of infection, recurrence of the contracture, secondary operation, and repaired area.

**Results:**

In 22 cases of extended transverse cervical flaps, 8 cases were embedded with two expanders, resulting in a total of 30 expanded flaps, which were successfully transferred to the neck and chest without necrosis at the distal end of compression, with good effect. Comparison of pedicled transverse cervical flaps with stepwise pressure packing and pedicled transverse cervical flaps alone revealed no significant difference in child satisfaction, the occurrence of infection, recurrence of the contracture, and secondary surgery (all *P* > 0.05). Yet, there was a significant difference in the repair area between two groups (*P* < 0.05).

**Conclusion:**

Prolongation of pedicled cervical flaps after expansion with stepwise pressure packing resulted in an effective method for repairing the large skin defect of children's face and neck caused by various diseases. In terms of increasing neck repair area, the operation with stepwise pressure dressing was significantly superior to the simple packing.

## Introduction

Congenital giant nevus, burn scars, and other lesions in neck and chest area may substantially affect the physical appearance, presenting a great source of stress to children and their parents. Currently, local and free flaps are commonly used in clinical surgery (1–3). With the application of expanded flaps, the area of defects repaired is increasing. One study showed that the area of anterior perforator of the transverse cervical artery flap to reconstruct neck defects could reach 300 cm^2^ (4). However, the skin and soft tissue in the donor site of children is not as much as that of adults, and too large expander will cause more damage (5–7). For pediatric huge neck defects, the pre-expansion of the cervical transverse flap is still insufficient. Therefore, we applied an extended predilated posterior cervical transverse flap with stepwise pressure dressing to repair large area of neck lesions in children, achieving good results.

## Materials and methods

### Patients

A total of 58 pediatric patients with neck and chest lesions were recruited from the Department of Plastic Surgery, Children's Hospital of Nanjing Medical University, between January 2011 to June 2021. Among them, 22 patients were treated with extended and pre-expanded transverse cervical flaps by stepwise compression dressing (14 males and 8 females, aged 4–12 years old), and 36 patients were treated with transverse cervical flaps alone (19 males and 17 females, aged 4–14 years old). The causes of skin defects in this study were: scars in 37 cases (20 cases with burn scar, 12 cases with poor results after scar excision and free skin grafting, 5 cases with hemangioma after radiotherapy or surgery) and congenital giant nevus in 21 cases. The course of the disease ranged from 0.5 to 8 years.

### Extra-long pre-expansion of the transverse cervical flap by stepwise compression dressing

**Stage I surgery**: The expander was inserted under general anesthesia, and the incision selection was performed as follows: the incision was mostly made on the shoulder, the midline of the sternum, or the lateral side of the chest wall. During the operation, dissection was carried out under the deep fascia, making sure not to damage the deep fascia. We made sure that the upper boundary of the dissection does not exceed the clavicle and the lower boundary does not exceed the breast. A total of 200–400 ml renal expander was used. An external injection pot was used; however, an external catheter needed a subcutaneous tunnel, usually placed under the incision. Five days after the operation, water injection was given twice a week, and each time water injection did not exceed 10% of the expanded capsule volume. The total water injection time was not less than 2.5–3 months, as too fast water injection might lead to skin elastic fiber fracture and skin “stretch marks” like changes. After adequate dilation, stage II surgery was performed at least 2 weeks later.

**Stage II surgery:** In the phase of preoperative design, the common carotid artery cervical transverse section of the cutaneous branches of the body surface projection point was determined according to the flap expansion (for the common carotid artery in sternocleidomastoid and transverse scapular hyoid muscle at the junction of the cutaneous branches of the fat into the neck triangle, about 1.8 cm on the clavicle middle, into the subcutaneous supraclavicular area, and outward and downward to separate two main artery skin nutrition supraclavicular areas under the skin). The lesion was removed and the size of the flap was drawn to be cut on the expanded skin according to the shape of the defect. The axial point of the flap was 1.8 cm above the midpoint of the clavicular bone at the posterior margin of the sternocleidomastoid muscle. The anterior edge of the trapezius muscle was taken as the posterior boundary of the flap, and the lateral boundary could reach the middle of the deltoid muscle. The medial boundary was the midline of the sternum, and our lower boundary could reach 6.0–8.0 cm below the nipple. It was 3–5 cm longer than the normal flap. During the operation, the outer, lower, and medial boundaries of the flap were dissected, and the tissue was removed to the clavicle level under the deep fascia. After crossing the clavicle, blunt separation was performed, and blood vessels entering the flap could be seen at this time. After separation to the pedicle, the separation depth of the flap rotation could cover the wound without tension. The flap could be rotated 90–180 degrees to cover the defect without considering the direction of the blood vessels. Then, the donor area was directly sutured. The incision was closed with 6-0 absorbable sutures. Finally, the stepwise pressure was exerted on the extra-long portion of the flap.

**Figure 1:**
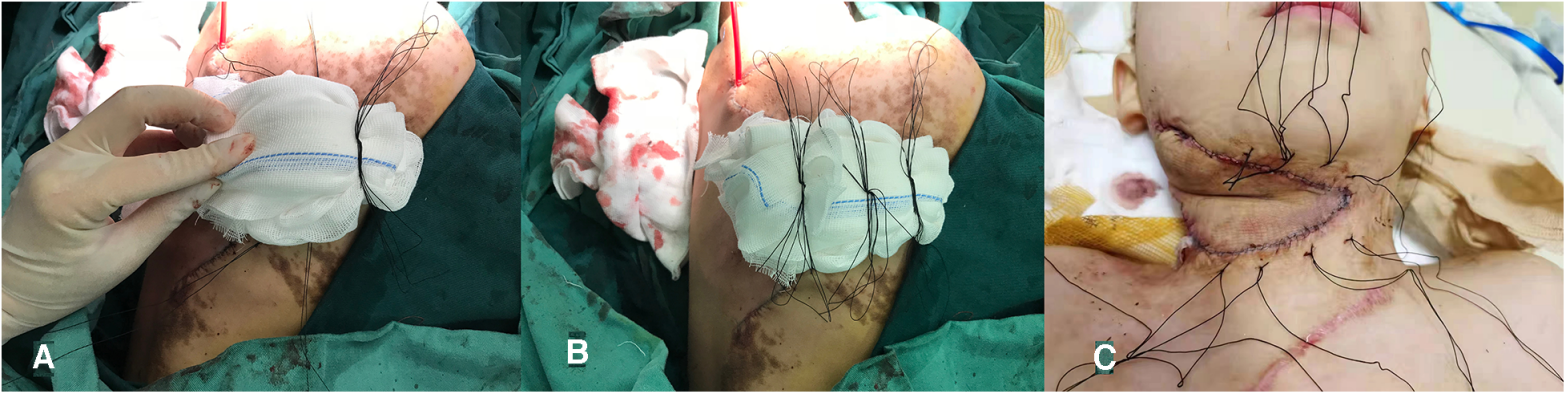
Stepwise pressure packing. (**A**) Apply pressure by applying a knot to the distal end of the flap. (**B**) Each knot has slightly less pressure than the previous one to create a pressure gradient. (**C**) The survival of the distal flap when the compression package was opened the next day.

**Figure 2:**
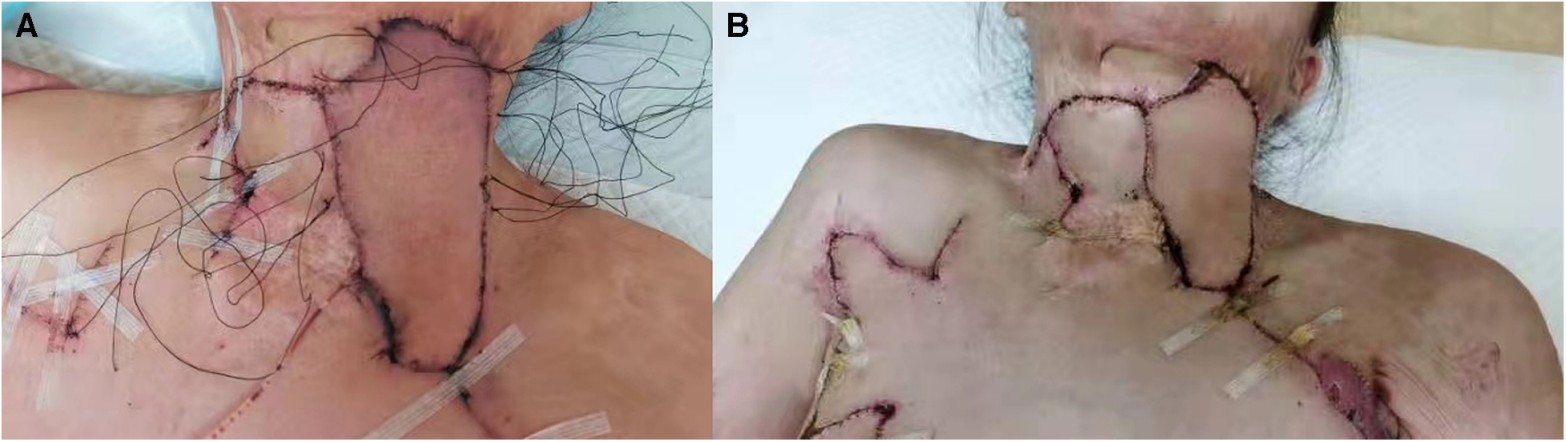
Applying the pressure pack again on the next day. (**A**) Applying pressure pack again on the next day. (**B**) The situation of flap and stitches removal when the package was opened for 7 days.

**Methods of Stepwise pressure packing** (Figure 1): After drainage, a compression knot was placed at the most distal end of the flap, followed by another knot at approximately 2–3 cm intervals toward the pedicle, for a total of approximately 3–4 knots. Each knot has slightly less pressure than the previous one to create a tension gradient. At last, the wound was wrapped with a dressing. After the operation, the patient was sent back to the ward. The next day, the compression bag was opened, and the color of the distal flap was observed. If the flap showed normal skin color, the compression bag was removed. If the color was blue or purple, a needle was used to bloodlet and the procedure of stepwise pressure dressing was repeated. After 7 days, the package was opened. Figure 2 shows the case of applying the pressure pack again the next day and the flap and stitches removal when the package was opened after 7 days.

The study was conducted in accordance with the Helsinki Declaration of 1975. All of the patients' guardians provided informed consent.

## Results

**Figure 3:**
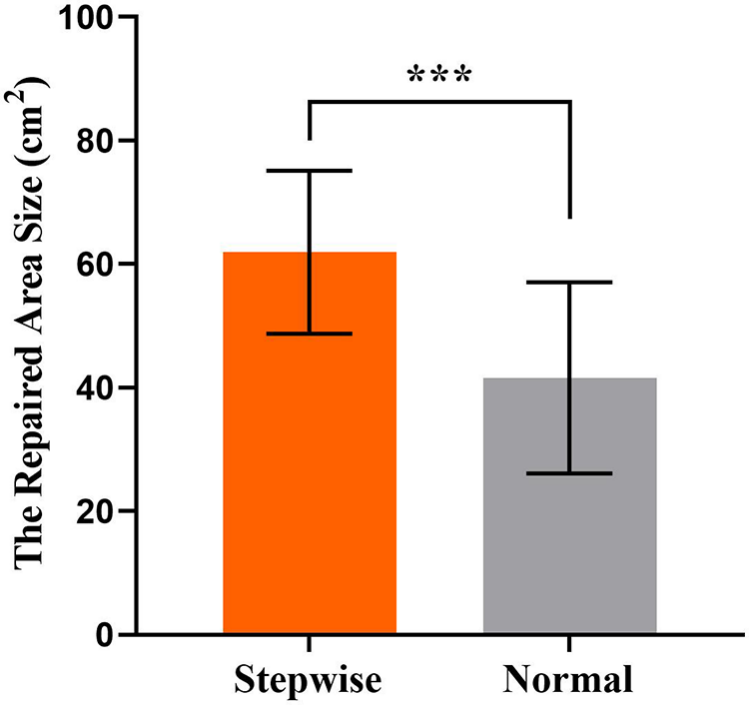
The area repaired by the transverse cervical flap was compared in the two groups. Results are mean ± SD. ***, *p* < 0.001. Stepwise, transverse cervical flap by stepwise compression dressing and Normal, transverse cervical flap by normal package.

This retrospective study included 58 patients, 22 of whom were treated with extended transverse cervical flaps with a stepwise compression dressing. A total of 30 expanders were embedded. The comparison of satisfaction, the occurrence of infection, incision dehiscence, recurrence of the contracture, and secondary operation between the two groups is shown in Supplementary Digital Content Supplementary Table S1. Incision infections were seen in 6 patients who received dilators, and 1 case with *Pseudomonas aeruginosa* infection was excluded (for this patient, the dilator was removed and the operation was terminated). The remaining infections were well controlled and qualified for stage II operation. Other complications included hematoma (5 case), dilator exposure (4 cases), recontracture (3 cases), incision dehiscence (3 case), and necrosis of flap tip (1 case). All the other patients successfully underwent the second stage of operation, and the color and texture of the transferred flap matched well with the normal skin of the adjacent part without contracture and pigmentation, resulting in a satisfactory effect. Comparison of pedicled cervical flaps with or without stepwise compression dressing show no difference in child satisfaction, the occurrence of infection, recurrence of contracture, and secondary surgery (all *P* > 0.05). Comparison of the area repaired by expanded flaps revealed significant differences between two groups (Figure 3).

### Example case

**Figure 4:**
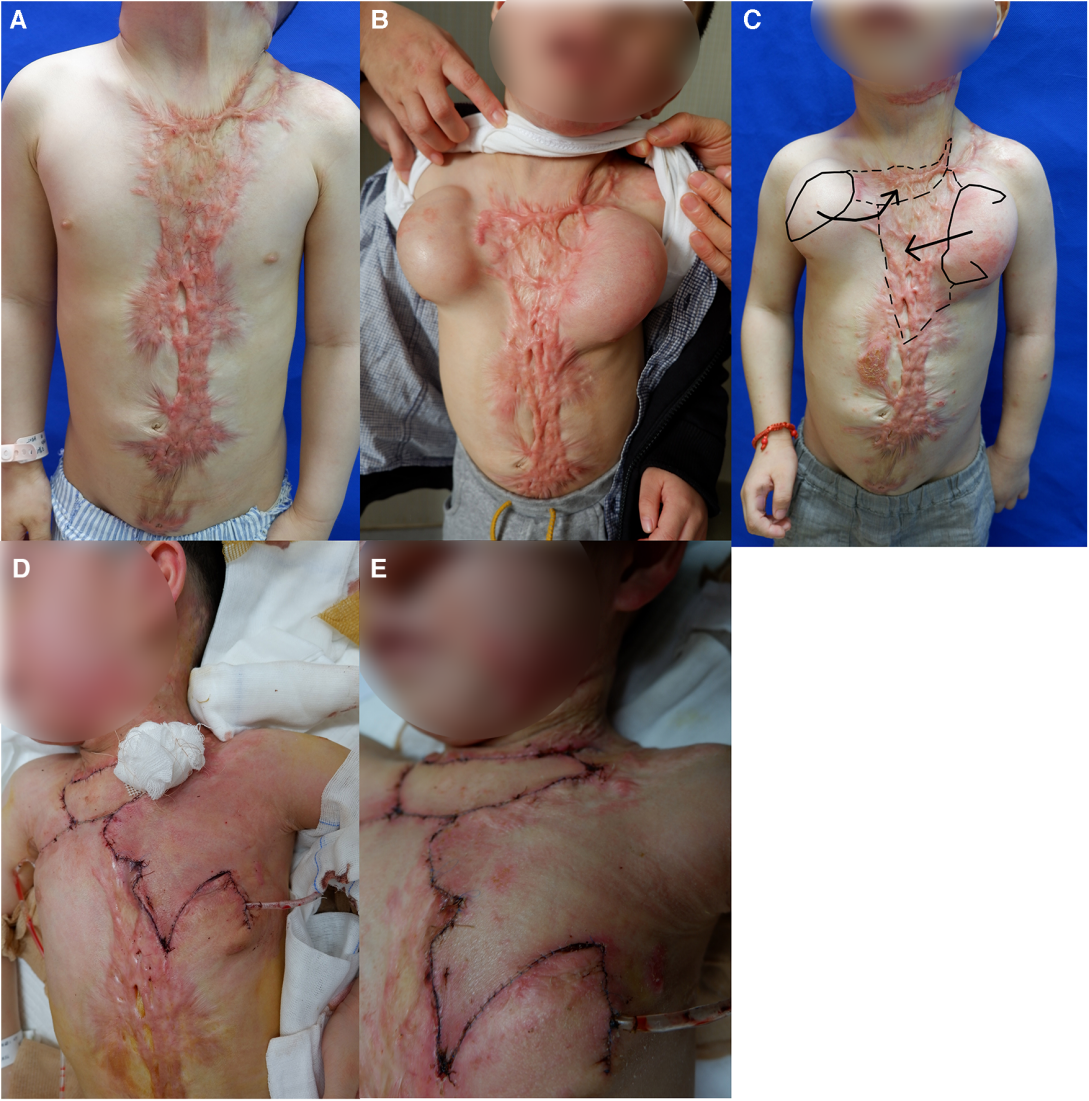
Photo of example cases. (**A**) A scar on the left neck and chest. (**B**) After the completion of the water injection expansion. (**C**) Surgical design. (**D**) Stepwise compression dressing method. **(E)** After the operation.

The 8 years old boy was admitted with A left neck and chest scar for 7 years. The patient suffered from an oral-cervicothorax burn 7 years ago, and the cervicothoracic scar contracture and cervicothoracic angle disappeared after the cure. Physical examination revealed a 16 cm × 15 cm scar on the left neck (Figure 4A), with limited neck extension. Next, a routine preoperative examination was performed, a 400 ml dilator and a 200 ml dilator were embedded in the chest area. After the operation, water injection was regularly administered in the outpatient department, and 4 months later, the water injection reached the rated capacity (Figure 4B). At this point, the second stage operation was performed, during which left neck scar was excised, and pre-expanded cervical transverse flap was transferred (surgical design is shown in Figure 4C) and repaired by stepwise compression dressing method (Figure 4D). The transverse cervical flap was 14 cm × 20 cm in size. After the operation, the skin flap survived, had good color and texture, and no obvious swelling (Figure 4E). A combined laser was sued postoperatively to prevent scar hyperplasia.

## Discussion

Free skin grafting is a commonly method for head and neck defects; however, this approach has a high recontracture rate and was phased out (1, 2). Based on the comparison of big data, Akita et al. (3) suggested that neck scar repair should be more effective with a skin flap, linear suture, or local skin flap repair. Stekelenburg et al. (8) argued that the perforator flap was more consistent with neck characteristics. Skin and soft tissue dilation have long been used to treat children.

At present, most existing reports focus on the study of its complication. In 2020, Wang et al. (9) reported a study of the related risk factors leading to the premature removal of children's dilators. Bjornson et al. (10) reported that complication in children using tissue dilators were as high as 40%, which could be reduced to 20% with careful operation and prevention. However, the literature reporting local neck predilation with an expander to treat neck defects is currently lacking. A preliminary study showed that when using anterior perforator of the transverse cervical artery (ap-TCA) flap to reconstruct intraoral defects, the flap could range from 6 × 4 cm to 15 × 9 cm (11). Chen et al. (4) used the ap-TCA flap to reconstruct neck defects in 11 patients, where the flap ranged from 12 × 8 cm to 15 × 20 cm. It was suggested that the ap-TCA flap and expanded ap-TCA flap could be considered reliable options for faciocervical deformities as they can be easily elevated and match well faciocervical area. Song et al. (12) reported that ap-TCA flap is one of the best options for cervicofacial reconstruction regarding color and texture match, with fewer flap complications. With regard to the expanded flap, the donor site can be directly sutured, leaving only an inconspicuous linear scar. Wang et al. (13) used a nonexpanded prefabricated ap-TCA flap for full facial reconstruction. Moreover, pre-expansion cervical transverse flap has advantages in repairing pediatric neck lesion. Repair involving the exposed and functional sites requires a similar skin tone and lower proneness to contracture in children. Accordingly, flap repair is an indubitably more reasonable approach. Nevertheless, the younger the child is, the less skin on the chest is inadequate. For the huge neck defects, the pre-expansion of the cervical transverse flap is still insufficient, as it cannot cover the whole neck defect. Therefore, we tried to extend the expanded flap with stepwise pressure dressing. Using this technique, the length of the original cervical transverse flap could be increased to ultra-long flap, so the repair distance could be elongated and the area could be enlarged.

It should be noted that the dilator should be placed on the sarcolemma of pectoralis major muscle during the first implantation. As the skin and soft tissue of children is thinner than that of adults, greater efforts should be made to try to avoid damage to the muscle membrane when stripping; otherwise, postoperative bleeding is more likely to occur. During the operation, the dilator should be fully stripped to the implanted area according to the plane size of the dilator so as to avoid the dilator folding into an angle, which was especially emphasized by Gosain et al. (14). During water injection, the expander moves down due to gravity. Elastic fabric can fix the lower edge of the expanded flap when the expander roughly expands. After the transverse cervical flap is fully predilated, a preoperative design should be carried out to determine the position of the transverse cervical artery crossing the fulcrum and protecting the artery pedicle. When rotating, the pedicle should be relaxed, not bloated, and the blood vessels should not be compressed. In addition, the defect can be drawn on a piece of gauze and turned to determine the size of the flap, after which the transfer can be simulated to the location of the defect. Next, it is necessary to determine the stepwise compression dressing area of the flap when doing the flap. The design should be super long, beyond the scope of the transverse neck flap. Also, the blood flow of the transverse neck flap should be observed. If purple color is observed or fading pressure shortly becomes purple again, then it is necessary to hit the stepwise pressure dressing.

Managing dilators in children is peculiar, as children need to be accompanied, and doctors should have enough patience. Older children are preoperatively informed of the treatment process and postoperative effects, which are then communicated to guardians required to accompany children. It is necessary to instruct guardians on how to observe the changes in the expanded flap during the water injection process and to disinfect the place where the aqueduct exits. Throughout the whole expansion cycle, children should abstain from vigorous exercise and be conveniently dressed to avoid external pressure. Postoperative nursing can affect the success of expansion and thus should not be ignored (15). Once the dilated skin is found to be red or increased in skin temperature, anti-infection treatment should be timely performed to prevent further aggravation of infection.

The postoperative care of the pre-expansion of the transverse cervical flap with the stepwise compression package should also be given attention. If the skin is dark purple when the compression dressing is opened on the second day, the arterial condition of the flap should be judged and the compression package should be continued until the flap color is normal.

In summary, we have demonstrated that prolongation of pedicled transverse cervical flap with stepwise compression dressing could notably increase the repair area without more complications and, therefore, is a safe and effective method for repairing the pediatric large neck skin defect.

## Data Availability

The original contributions presented in the study are included in the article/**Supplementary Material**, further inquiries can be directed to the corresponding author.
