# Supplementary material for: Repair of cervicothoracic skin defects with extra-long transverse cervical flaps by stepwise pressure packing in children: a technical innovation
Source: Front Pediatr. 2023 Nov 21;11:1269695. doi: 10.3389/fped.2023.1269695 (PMC10699302; doi:10.3389/fped.2023.1269695)
Supplement: Supplementary file 1 [file Table1.doc]

Supplemental Table

Supplementary Digital Content Table 1 Comparison of transverse cervical flap with or without stepwise compression dressing

|  | transverse cervical flap by stepwise compression dressing（22） | transverse cervical flap （36） |
| --- | --- | --- |
| Children with satisfaction | 21 | 34 |
| infections | 2 | 3 |
| Incision dehiscence | 1 | 2 |
| Necrosis of flap tip | 0 | 1 |
| Contracture reoccurs | 1 | 2 |
| Secondary surgery | 1 | 2 |

P>0.05，Not significant.
